# Effect of Electroacupuncture on Bladder Dysfunction via Regulation of MLC and MLCK Phosphorylation in a Rat Model of Type 2 Diabetes Mellitus

**DOI:** 10.1155/2021/5558890

**Published:** 2021-06-10

**Authors:** Xuke Han, Yang Gao, Xuan Yin, Shengju Wang, Xiaoran Zhang, Qiu Chen

**Affiliations:** ^1^Hospital of Chengdu University of Traditional Chinese Medicine, Chengdu, Sichuan 610072, China; ^2^Chengdu University of Traditional Chinese Medicine, Chengdu, Sichuan 610075, China; ^3^Shanghai Municipal Hospital of Traditional Chinese Medicine, Shanghai University of Traditional Chinese Medicine, Shanghai 200071, China

## Abstract

Previous studies observed have reported that electroacupuncture (EA) is effective in relieving diabetic bladder dysfunction (DBD); however, little is known about the mechanism. Therefore, we explored the effects and mechanisms of EA on DBD in streptozotocin–high-fat diet- (STZ–HFD-) induced diabetic rats. The Sprague-Dawley male rats were divided randomly into four groups: normal group, diabetes mellitus group (DM group), DM with EA treatment group (EA group), and DM with sham EA treatment group (sham EA group). After 8 weeks of EA treatment, the body weight, serum glucose, bladder weight, and cystometrogram were evaluated. The bladder wall thickness was examined by abdominal ultrasound imaging. After the transabdominal ultrasound measurements, hematoxylin-eosin (HE) staining was used to observe the bladder mucosa layer. The bladder detrusor smooth muscle cells (SMCs) and fibroblasts were observed under transmission electron microscopy (TEM). The phospho-myosin light chain (p-MLC), phospho-myosin light chain kinase (p-MLCK), and phospho-myosin phosphatase target subunit 1 (p-MYPT1) levels in the bladder were examined using Western blot. The bladder weight, serum glucose, bladder wall thickness, volume threshold for micturition, and postvoid residual (PVR) volume in the diabetic rats were significantly higher than those in the control animals. EA treatment significantly reduced the bladder weight, bladder wall thickness, volume threshold for micturition, and PVR volume in diabetic rats. EA caused a significant increase in the MLC dephosphorylation and MLCK phosphorylation levels in the group compared to the sham EA and model groups. EA reduced the infiltration of inflammatory cells in the bladder mucosa layer of diabetic rats. In addition, EA repaired the damaged bladder detrusor muscle of diabetic rats by reducing mitochondrial damage of the SMCs and fibroblasts. Therefore, EA could reduce the bladder hypertrophy to ameliorate DBD by reversing the impairment in the mucosa layer and detrusor SMCs, which might be mainly mediated by the regulation of p-MLC and p-MLCK levels.

## 1. Introduction

Diabetes mellitus (DM) as a metabolic disease has reached epidemic proportions worldwide and is associated with various complications, including retinopathy, neuropathy, and nephropathy [1]. The global prevalence of diabetes in adults has reached 9.3%; that is, there are already 463 million adult diabetic patients worldwide [2]. Approximately 80% of type 2 DM (T2DM) patients have urinary system symptoms, including urinary retention, incontinence, frequency, and urgency [3]. Diabetic bladder dysfunction (DBD), one of the major sequelae of DM, is characterized by decreased bladder detrusor contractility and disturbance of bladder activity, which in turn manifests as increased bladder capacity (BC) and residual urine volume. Previous studies have shown that 50% of diabetic patients experience DBD-related symptoms after 10 years of DM progression [4], and current Western medicine treatments cannot effectively improve these symptoms [5]. Therefore, it is of great significance to find a safe and effective treatment for DBD.

The level of myosin light chain (MLC) phosphorylation is an important determinant of smooth muscle contraction. The phosphorylation of MLC primarily regulates the force production and maintenance in the smooth muscle [6]. Myosin light chain phosphatase (MLCP) activity is associated with mammalian bladder function, and it is speculated that a change in MLC may play an important role in the nervous and myogenic control of the bladder [7]. The function of striated or smooth muscle is regulated by the balance of myosin light chain kinase (MLCK) and MLCP activity. It is suggested that the phosphorylation of MLC catalyzed by Ca^2+^-calmodulin- (CAM-) dependent MLCK and dephosphorylation catalyzed by MLCP help regulate the contraction and relaxation of smooth muscle [8, 9]. The phosphorylation of smooth muscle regulatory light chain by MLCK is the basic requirement for smooth muscle contraction and hollow organ physiological activities [10]. It is generally believed that sustained contraction mainly occurs through Ca^2+^ sensitization. When the Ca^2+^ concentration continuously decreases, the smooth muscle contraction can be maintained by inhibiting the activity of MLCP [11]. The myosin phosphatase target subunit 1 (MYPT1) in the smooth muscle is one of the myosin phosphatase-targeting subunits of MLCP [12]. The underlying mechanism of sustained contraction is related to MYPT1 [13]. Multiple lines of evidence imply that MYPT1 may regulate the biochemical activity of MLCP through multiple mechanisms, thereby regulating MLC phosphorylation and affecting bladder smooth muscle detrusor contraction [14]. These results suggest that the pathogenesis of DBD may be closely related to MLC phosphorylation.

Acupuncture has been used to treat DM for thousands of years, and its curative effect has been confirmed by many clinical pieces of evidence and animal studies [15]. It is reported that acupuncture is effective in improving urodynamic function and various symptoms related to DBD, and thus acupuncture may be a novel form of alternative therapy for the radical treatment of DBD [16]. Previous studies have also revealed the advantages of acupuncture in the treatment of bladder dysfunction, which includes alleviation of symptoms (improvement of chronic urinary retention and incontinence), improvement in the quality of life of patients, and delayed progression of DBD [17]. Electroacupuncture (EA) is based on conventional acupuncture treatment, wherein an electric current is passed through a specific electrical device to stimulate the body's acupoints. In clinical trials, BL33 (Zhongliao) and SP6 (Sanyinjiao) are the two most commonly used acupoints to treat bladder dysfunction [18]. In addition, a related animal study has shown that a 2-Hz EA stimulation at BL33 can enhance the detrusor smooth muscle contraction and improve voiding dysfunction [18]. From a neurophysiological perspective, the muscle tissues at BL33 and SP6 are dominated by the same segment (S2–S4) of the spinal cord micturition center [19]. Moreover, BL33 is located at the third posterior sacral foramen. The deep insertion of EA at BL33 can directly stimulate the sacral nerve, which may promote the rhythmic contraction of the detrusor and bladder sphincter, increase their coordination function, and facilitate the formation of urination reflex [20, 21]. EA has been shown to effectively inhibit overactive bladder in rats, improve bladder compliance, reduce the pathological damage of bladder tissue, and improve bladder function [22, 23]. Therefore, we performed EA interventions on the BL33 and SP6 acupoints. Previous studies have also found that the phosphorylation level of MLC in diabetic animals was significantly higher than that of the control group, thereby suggesting that the regulation of MLC phosphorylation may play a role in the detrusor contractility and bladder dysfunction caused by diabetes [24]. Based on previous studies, we propose a hypothesis that the mechanism of EA in the treatment of DBD may be attributed to the regulation of the bladder contraction element and MLC phosphorylation levels to promote detrusor relaxation and ultimately improve bladder function. In this study, we investigated the effects of EA to improve bladder function, histomorphological changes, and molecular changes using a streptozotocin–high-fat diet- (STZ–HFD-) induced T2DM rat model. Comprehensive application of cystometrogram, transabdominal ultrasound measurement, hematoxylin-eosin (HE) staining, transmission electron microscopy (TEM) examination, and Western blot was used to explore the effect of EA on DBD and its possible biological mechanism from different levels of function, morphology, and molecule.

## 2. Materials and Methods

### 2.1. Animal Model

A total of 50 male Sprague-Dawley rats, aged 13–15 weeks and weighing 180–220 g, were obtained from the Chengdu Dossy Experimental Animals Company (China). The rats were kept under standard conditions (21 ± 2°C and 40–70% relative humidity, with a 12 h light-dark cycle; standard laboratory chow and tap water were available ad libitum).

After one week of adaptive feeding, the rats were randomly divided into a control group (*n* = 10) and a model group (*n* = 40). The model rats were fed with HFD for 4 weeks to induce insulin resistance. After 24 h of fasting, the diabetic model rats were induced by a single intraperitoneal injection of STZ (60 mg/kg) diluted in 0.1 mol/L citrate buffer solution [25]. After 48 hours, the fasting serum glucose concentration was measured, and 30 rats were diagnosed with DM (fasting serum glucose > 12 mmol/L). These rats were then assigned into three groups: (1) DM group (*n* = 10), (2) DM with EA treatment group (EA group; *n* = 10), and (3) DM with sham EA treatment group (sham EA group; *n* = 10). After 8 weeks of intervention, the body weight, serum glucose, and bladder weight of the rats were tested. All rats underwent cystometry and transabdominal ultrasound to examine the bladder function at week 12. HE staining was used to observe the state of bladder mucosa layer. The morphological features of the bladder detrusor smooth muscle cells (SMCs) and fibroblasts were evaluated under TEM. The expressions of p-MLC, p-MLCK, and p-MYPT1 were measured using Western blot.

All animal experimental procedures were performed in accordance with the National Institute of Health guidelines for the Care and Use of Laboratory Animals (China). The study has also been approved by the Hospital of Chengdu University of Traditional Chinese Medicine (Chengdu, China).

### 2.2. Experimental Design

Rats in the EA group were treated with EA for 8 weeks. Acupuncture points were located according to “the map of acupuncture points for rats” in Experimental Acupuncture Science. SP6 is located 10 mm above the tip of the medial malleolus in rats. Since rats have only three pairs of posterior sacral foramina, we located the BL33 point at the second posterior sacral foramen [18, 26]. Disposable sterile stainless steel acupuncture needles (0.25 mm × 25 mm; Cofoe Medical Technology Co., Ltd., Hunan, China) were inserted bilaterally at BL33 to a depth of approximately 15 mm and vertically inserted at SP6 to a depth of approximately 3 mm bilaterally. The *E* Stimulator, a Hua Tuo Acupoint Nerve instrument (SDZ-V; Hua Tuo Medical Technology Co., Ltd., Suzhou, China), was connected to the acupuncture needles at all acupoints. EA stimulation parameters were as follows. Continuous wave frequency was 2 Hz. The intensity was set to 0.1 mA and increased to 10 mA, resulting in mild limb trembling, which was tolerated by the rats. The treatment was performed once a day for 8 consecutive weeks, and each session lasted 20 minutes.

According to our previous animal experimental protocol [27] and related clinical studies [28], acupuncture needles were affixed to the surface of BL33 and SP6 in the sham EA group, without penetrating into the acupoints in order to avoid nonspecific acupuncture effect. The stimulator is connected to the acupuncture needles, but the power switch is turned off. The needle retention time and intervention duration in the sham EA group were the same as those of the EA group. Rats in the normal and DM groups did not receive any treatment, but rats in the DM group were fed with HFD for 8 weeks.

### 2.3. Cystometrogram

After 8 weeks of intervention, the rats (*n* = 8 per group) were anesthetized with sodium pentobarbital (40 mg/kg, intraperitoneal). A polyethylene tube (PE-50, Clay Adams, New Jersey, USA) was inserted into the bladder through the urethra and connected to a pressure transducer (Harvard Apparatus, Massachusetts, USA) and a microinjection pump (PHD 22/2000 Syringe Pump, Harvard Apparatus, Massachusetts, USA) via a 3-way stopcock, to record the bladder pressure and infuse the bladder with normal saline. The micturition volume was recorded by a liquid collector (Isometric Force Transducer, Harvard Apparatus, Massachusetts, USA). Saline was infused into the bladder at a rate of 0.6 ml/min. The urodynamic parameters of each group, such as volume threshold for eliciting micturition, maximum intravesical pressure (Pv, max), BC, voided volume, postvoid residual (PVR) volume, and *V*% (100 × PVR/BC), were calculated and compared. Measurements in each rat represented the average of five micturition cycles after an initial 45 min stabilization period.

### 2.4. Ultrasonography

After the cystometrogram was completed, bladder ultrasonography was used to assess the bladder wall thickness. Before performing bladder transabdominal ultrasound measurements, the rat's abdominal hair was shaved. After the bladder was emptied, approximately 1.5 ml of normal saline was injected into the rat's bladder with a microinjection pump. The rats were placed in a supine position, and bladder images were recorded with a hand-held probe. For image and data acquisition, a clinical research ultrasound system (MyLab™Six, Esaote, Genoa, Italy) and a clinically compatible linear array ultrasound transducer were used. Changes in bladder volume and bladder imaging, such as shape, size, bladder wall status, and abnormal echo, were detected.

### 2.5. HE Staining

After the transabdominal ultrasound measurements, the rats (*n* = 8, per group) were anesthetized with sodium pentobarbital (40 mg/kg, intraperitoneal) and decapitated. The whole bladder was weighed. A portion of the bladder was fixed in a 10% neutral formaldehyde solution for 24 h and then embedded in paraffin. The bladder tissue sections were stained with HE stain. The gross appearance was observed in high-power (×100) fields by examining each section, and specific pathological changes were then observed in higher power (×400) fields. The rest of the bladder tissue was frozen in liquid nitrogen and stored at −80°C.

### 2.6. TEM Examination

The bladder tissues were isolated in three 0.5 mm sized chunks and fixed in 4% glutaraldehyde and 1% osmium tetroxide for 2 h. The tissues were then subjected to gradient dehydration by acetone, and the concentration of dehydrating agent used was 30%⟶50%⟶70%⟶80%⟶90%⟶95%⟶100%. The three samples were placed in a mixture of acetone and epoxy resin (3 : 1, 1 : 1, and 1 : 3) for 60 min and infiltrated overnight in a mixture of acetone and embedding agent. A pure embedding medium was poured into the embedding board, which formed a solid matrix by thermal polymerization. An ultrathin microtome was used to cut the bladder samples into 50 nm sections. The samples were stained with uranium acetate (15 min) and lead citrate (2 min) at room temperature. Finally, TEM (JEM-1400PLUS, JEOL, Ltd., Tokyo, Japan) was used to perform image analysis.

### 2.7. Western Blot

The bladder tissue proteins were prepared using the Total Protein Extraction Kit (Beyotime Biotechnology, Jiangsu, China), and the protein concentration was determined using a Pierce® BCA Protein Assay Kit (Thermo Fisher Scientific, Waltham, Massachusetts, USA). Membrane proteins (30 *μ*g) were fractionated by electrophoresis through a 10% sodium dodecyl sulfate-polyacrylamide gel electrophoresis and then transferred onto polyvinylidene difluoride membranes (Sigma-Aldrich, Missouri, USA). Membranes were blocked with TBST (1 × Tris-buffered saline, 0.1% Tween 20 with 5% nonfat dry milk) for 1 h and then incubated with primary antibodies overnight at 4°C. The concentrations of primary antibodies were as follows: p-MLC (1 : 1000, Abcam), p-MLCK (1 : 1000, Abcam), p-MYPT1 (1 : 1000, Affinity), and *β*-actin (1 : 10000, ABclonal). After overnight incubation, the membranes were washed three times with TBST for 5 min each and then incubated with horseradish peroxidase-conjugated secondary antibodies for 3 h at room temperature, followed by three more washes with TBST for 10 min each. Finally, protein bands were detected with the enhanced chemiluminescence kit (Affinity Biosciences, Ohio, USA). Chemiluminescent signals were detected and analyzed by the ChemiDoc XRS imaging system (Bio-Rad, Hercules, California, USA).

### 2.8. Statistical Analysis

All data were statistically analyzed by SPSS Statistics version 17.0 for Windows 10. The results were compared between different groups by the one-way analysis of variance, followed by the Student–Newman–Keuls q-test and expressed as mean ± standard error of the mean. *P* ≤ 0.05 was considered statistically significant.

## 3. Results

### 3.1. Body Weight, Serum Glucose, and Bladder Weight

**Figure 1:**
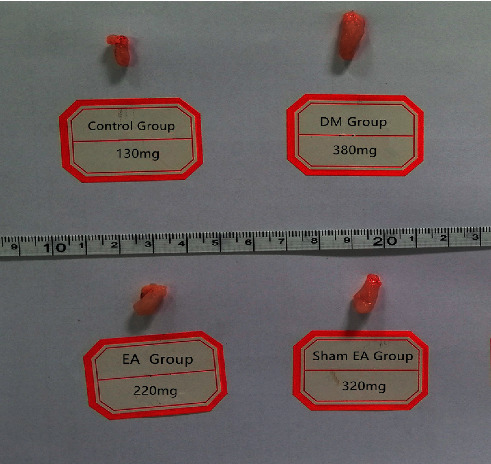
A sample of bladder weight measurement.

After the 8-week intervention, significant hyperglycemia and decreased body weight were observed in all diabetic rats in the DM group (20.6 ± 2.4 mmol/L, 225.7 ± 10.78 g), EA group (20.9 ± 2.6 mmol/L, 226.5 ± 9.5 g), and sham EA group (20.5 ± 3.2 mmol/L, 225.1 ± 11.6 g) compared to those in the control group (3.64 ± 0.25 mmol/L, 386.4 ± 12.5 g; *P* < 0.01). The bladder weight of rats in the DM group (368 ± 29.5 mg), EA group (216 ± 18.6 mg), and sham EA group (331 ± 25.6 mg) was significantly (*P* < 0.05) greater than that of the control group rats (120 ± 19.7 mg). However, the bladder weight of diabetic rats in the EA group significantly decreased compared to the sham EA group (*P* < 0.05). There was no significant difference between the sham EA group and the DM group. EA and sham EA treatment did not have any effect on the body weight and serum glucose levels throughout the experiment (Table 1). A sample of bladder weight measurement is shown in Figure 1.

### 3.2. Cystometric Parameters

**Figure 2:**
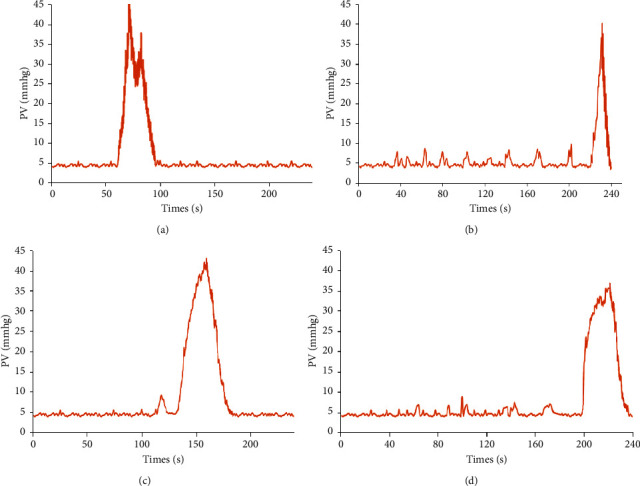
Typical urodynamic curves of cystometrogram of rats in each group. (a) Control. (b) DM. (c) EA. (d) Sham EA. The typical recordings of cystometrogram of the DM (b) and sham EA (d) group indicate that the threshold of micturition was significantly delayed, and there were some unstable spontaneous contractions before micturition compared to the other groups. DM: diabetes mellitus; EA: electroacupuncture

The volume threshold for micturition, PVR volume, and V% in the DM group were significantly (*P* < 0.05) higher than those in the control group. EA treatment significantly (*P* < 0.05) decreased the volume threshold for micturition, PVR volume, and V% in the DM group compared to those of the sham EA group. Furthermore, there was no significant difference in Pv, max among the four groups (Table 2; Figure 2).

### 3.3. Ultrasonography

**Figure 3:**
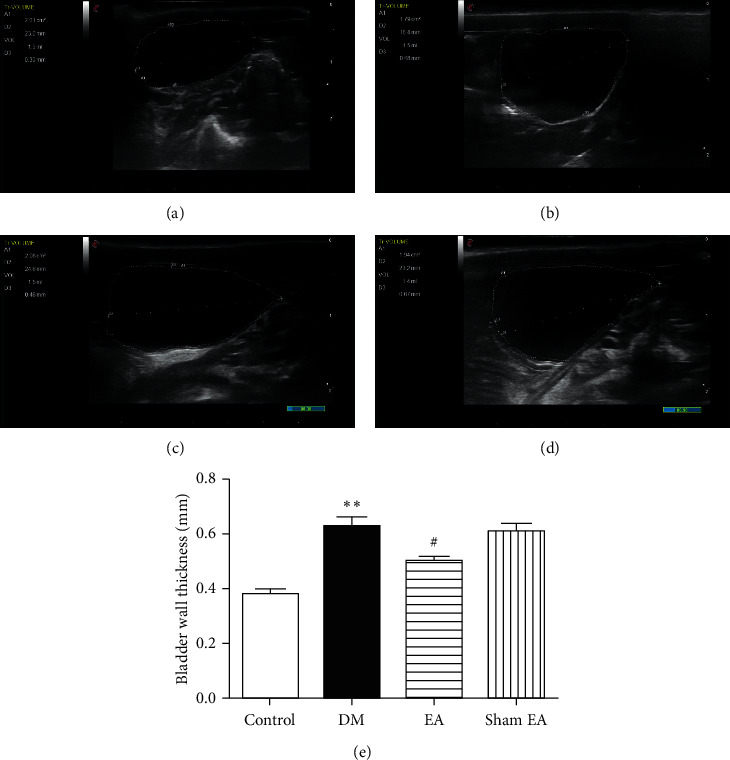
Ultrasonography test in rats after the 8-week intervention bladder ultrasound images of the control group (a), DM group (b), EA group (c), and sham EA group (d). The results of bladder wall thickness (e) in the ultrasonography test. Data were expressed as mean ± SEM. ^*∗∗*^*P* < 0.01 compared to the control group; ^#^*P* < 0.05 compared to the sham EA group. DM: diabetes mellitus; EA: electroacupuncture; SEM: standard error of the mean.

The bladder wall thickness was significantly increased in the DM group compared to the control group (0.71 ± 0.244 versus 0.37 ± 0.18, *P* < 0.01). There was no significant difference between the DM and sham EA group. After 8 weeks of treatment, the bladder wall thickness was significantly decreased in the EA group compared to the sham EA group (0.52 ± 0.109 versus 0.68 ± 0.125, *P* < 0.05; Figure 3).

### 3.4. Histological Findings

**Figure 4:**
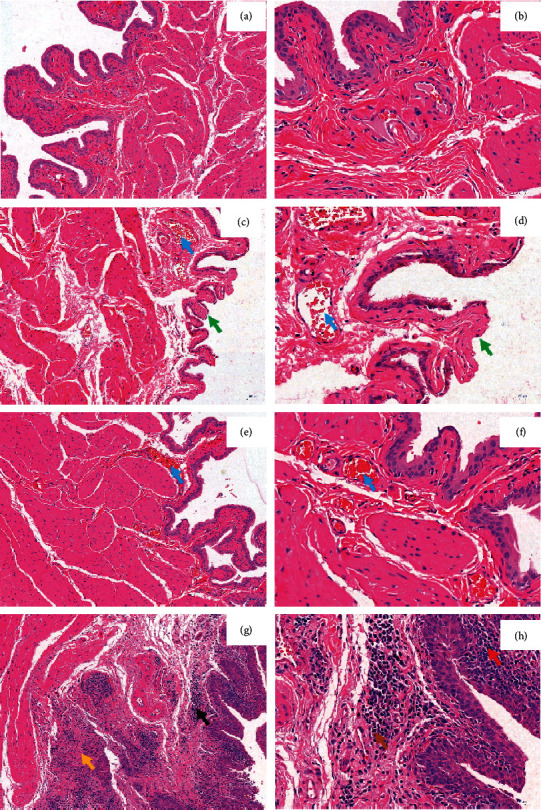
Hematoxylin-eosin (HE) staining. Control group: (a) (×100) and (b) (×400); DM group: (c) (×100) and (d) (×400); EA group: (e) (×100) and (f) (×400); and sham EA group: (g) (×100) and (h) (×400). The blue arrowheads indicate hyperemia of bladder mucosa cell; green arrowheads indicate the necrosis of bladder mucosa cell; yellow arrowheads indicate fibrous tissue hyperplasia of the bladder mucosa layer; black arrowheads indicate the inflammatory cell infiltration of bladder mucosa layer; brown arrowheads indicate lymphocytes; and red arrowheads indicate plasma cells.

The DM group showed an increased bladder mucosal cell denaturation, necrosis, and hyperemia in the bladder mucosa layer as compared to the control group. The sham EA group showed increased inflammatory cell infiltration and fibrous tissue hyperplasia in the bladder mucosa layer as compared to the EA group. After 8 weeks of EA intervention, the infiltration of inflammatory cells in bladder mucosa was reduced, and only a few cells were hyperemic (Figure 4).

### 3.5. TEM Examination

**Figure 5:**
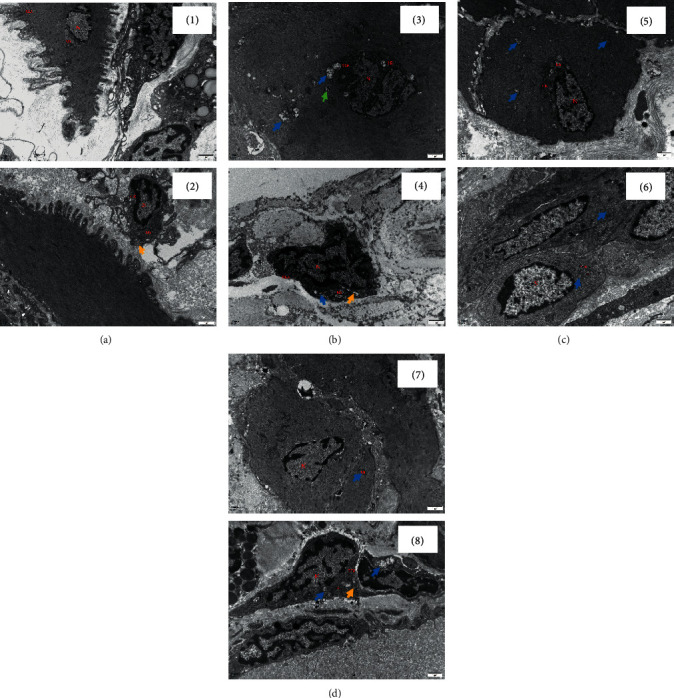
Transmission electron microscopy (TEM) of the rat bladder detrusor. (a) Control group. (b) DM group. (c) EA group. (d) Sham EA group. Organelles such as nucleus, Mi, and RER are seen. (1) A representative image from the normal bladder SMCs at a magnification of ×12,000. (2) A representative image from the normal bladder fibroblasts at a magnification of ×12,000. The yellow arrowheads indicate the expansion of RER. (3) A representative image from the bladder SMCs of diabetic rats at a magnification of ×12,000. The blue arrowheads indicate the swelling of Mi, and the green arrowheads indicate Mi autophagy. (4) A representative image from the bladder fibroblasts of diabetic rats at a magnification of ×12,000. The yellow arrowheads indicate the expansion of RER, and the blue arrowheads indicate the mild swelling of Mi. (5) A representative image of bladder SMCs in diabetic rats treated with sham EA, at a magnification of ×12,000. The blue arrowheads indicate the mild swelling of Mi. (6) A representative image of bladder fibroblasts in diabetic rats treated with sham EA, at a magnification of ×12,000. The yellow arrowheads indicate the dilatation of RER, and the blue arrowheads indicate the swelling of Mi. (7) A representative image of bladder SMCs in diabetic rats treated with EA, at a magnification of ×12,000. The blue arrowheads indicate the swelling of Mi. (8) A representative image of bladder fibroblasts in diabetic rats treated with EA, at a magnification of ×12,000. The blue arrowheads indicate the mild swelling of Mi. Mi: mitochondria; RER: rough endoplasmic reticulum; EA: electroacupuncture.

The ultrastructure of bladder detrusor was essentially normal in the control group. The nucleus of SMCs and fibroblasts had an irregular polygonal shape, the chromatin was evenly distributed (mainly euchromatin), and the nuclear membrane was clear and complete. Organelles such as mitochondria (Mi), rough endoplasmic reticulum (RER), and ribosomes were observed in the cytoplasm and between muscle fibers, with complete and clear structures. Occasionally, the RER expanded into a cystic shape (Figure 5(a)).

In the DM group, most of the Mi in SMCs were swollen; cristae were broken, dissolved, or even disappeared; and a small amount of autophagy was observed. In the fibroblasts, swelling of a few Mi was observed, the cristae were broken, dissolved, or even disappeared, and a few RER appeared cystic (Figure 5(b)).

In the EA group, some Mi were swollen and the cristae were broken, dissolved, or even disappeared in SMCs. Mild swelling of some Mi was observed in the fibroblasts (Figure 5(c)).

In the sham EA group, mild swelling of a small number of Mi was observed in SMCs. In the fibroblasts, most of the Mi were swollen; the cristae were broken, dissolved, or even disappeared; and some RER showed slight expansion (Figure 5(d)).

### 3.6. Western Blot

**Figure 6:**
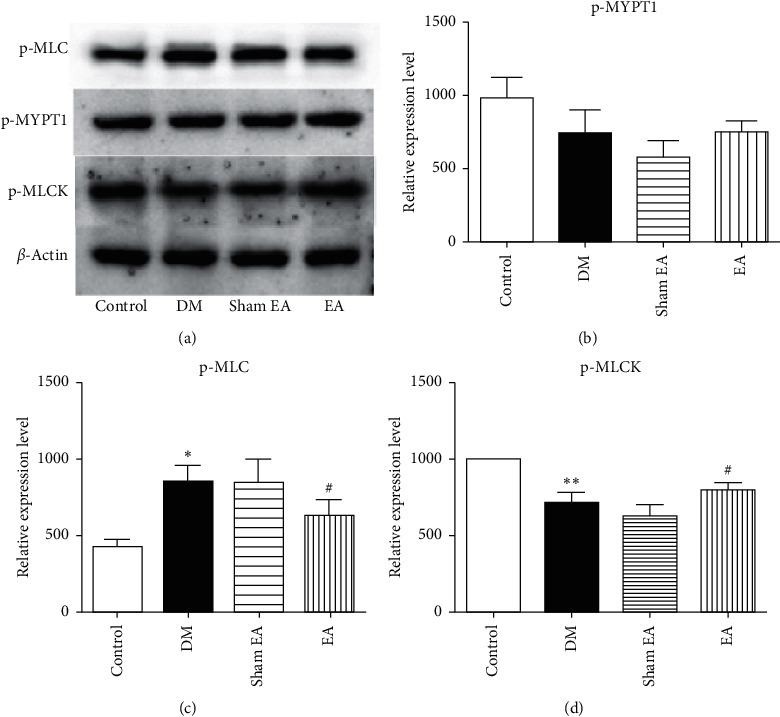
Western blot bands of expression of p-MLC, p-MLCK, and p-MYPT1 protein in rats after the 8-week intervention. Data were expressed as mean ± SEM. ^*∗*^*P* < 0.05 compared to the control group; ^*∗∗*^*P* < 0.01 compared to the control group;^#^*P* < 0.05 compared to the sham EA group. p-MLC: phospho-myosin light chain; p-MLCK: phospho-myosin light chain kinase; p-MYPT1: phospho-myosin phosphatase target subunit 1; SEM: standard error of the mean; EA: electroacupuncture.

The p-MLC level was significantly increased in the DM group compared to the control group (*P* < 0.05). It was reduced after EA treatment as compared to the sham EA group (*P* < 0.05). The expression of p-MLCK protein was significantly lower (*P* < 0.01) in the DM group than in the control group. The p-MLCK protein level was significantly increased in the EA group compared to the sham EA group (*P* < 0.05). No differences were found in p-MYPT1 comparisons among all the groups (Figure 6).

## 4. Discussion

The present study focused on the bladder dysfunction of a T2DM rat model. An STZ–HFD-induced diabetic rat model is suitable to study the complications of T2DM and determine the antidiabetic treatment, and it has been proved as the most appropriate model [29–31]. Because the STZ–HFD model can simulate insulin resistance through islet destruction, reflecting the typical pathogenesis of T2DM, it is also considered to be the best T2DM rat model [32, 33]. In addition, this method only takes approximately 2 months to obtain the insulin resistance model, which greatly saves time and cost compared with the transgenic or genetic model [34]. T2DM influences the patients' bladder function and produces neurogenic and myogenic lesions, leading to various bladder dysfunctions, such as urgency, frequency, incontinence, dysuria, and urinary retention [35]. Similar changes are observed in the STZ–HFD-induced diabetic model rats [36, 37]. Our findings indicate that the body weight of the STZ–HFD-induced T2DM model rats (DM group) was significantly decreased and the serum glucose and bladder weight were increased compared with the control group rats. The data were consistent with the current study, which shows that an STZ injection and HFD could induce T2DM symptoms, illustrating that STZ–HFD model rats could effectively imitate the symptoms of T2DM in patients [36].

Previous studies have found that DBD symptoms can appear 2-3 months after STZ administration [38]. It is speculated that, in a type 1 DM model, the compensatory state of DBD occurred 1-2 months after STZ administration, including increased voided volume, bladder weight, PVR volume, and *V*%. A relevant study that used STZ–HFD to induce a T2DM rat model found that this model developed a compensated state (overactive bladder symptoms) at 1 week after STZ administration and a decompensated state (underactive bladder) at 4 months [39]. Their results demonstrated that the early stage of DBD can be characterized by an increased blood glucose level and bladder wet weight. Moreover, this state appeared 1 week after the STZ injection and lasted 3 months. Currently, research on DBD is mostly carried out in the eighth week after the successful establishment of a diabetic rat model [40, 41]. Therefore, in this study, STZ–HFD was selected to induce T2DM, and the evaluation was conducted after 8 weeks of feeding. The results showed that the bladder weight, bladder wall thickness, and PVR volume increased, proving that this method can effectively replicate the symptoms of DBD. These findings confirm that the STZ–HFD model of DBD exhibits bladder dysfunction-related indicators.

EA has been widely accepted as an alternative therapy for various diseases [42]. The efferent signal from the spinal micturition center is sent to the intramural ganglion of the bladder wall along with the pelvic nerve, which can cause contraction of the bladder detrusor smooth muscle [43, 44]. Relevant studies have found that acupuncture can activate the afferent nerve fibers of the dorsal root of the spinal cord [45]. Since the micturition center of BL33 and SP6 is located in the same segment, EA at these acupoints may activate sacral afferent nerve fibers to enhance bladder detrusor smooth muscle contraction [46]. Previous studies have reported that an increased bladder weight is correlated with increased bladder threshold capacity and residual urine [47]. In this study, the bladder weight was measured to determine whether bladder hypertrophy occurred in T2DM rats. The bladder weights of the DM group were significantly increased compared with the control group. Our results are consistent with those of previous studies in that the volume threshold for micturition, PVR volume, and V% in the DM group were significantly increased compared with the control group. Importantly, bladder weight in the EA group was significantly decreased compared to the sham EA group. After 8 weeks of EA treatment, cystometrogram revealed that the delay of the volume threshold for micturition was significantly improved and PVR volume and *V*% were significantly decreased compared with the sham EA group, suggesting that EA treatment could restore the micturition reflex. In order to further evaluate the lesions of the bladder, we used transabdominal ultrasound measurements to detect the thickness of the bladder wall. We observed that the bladder wall of the DM group was significantly thicker than that of the control group. This is mainly due to bladder detrusor smooth muscle layer hypertrophy [48]. Furthermore, the bladder wall thickness of the EA group was significantly lower than that of the sham EA group. These results suggest that EA at BL33 and SP6 can reduce the bladder wall thickness, reduce bladder hypertrophy, and regulate the contractile function of the bladder detrusor smooth muscle to restore micturition reflex.

Currently, it is generally believed that the contraction of bladder detrusor smooth muscle is a Ca^2+^- and ATP-dependent process. The MLC phosphorylation process decomposes enough ATP, converts chemical energy into mechanical energy, and causes smooth muscle contraction through the myosin-affecting actin [49, 50]. Therefore, the higher the degree of MLC phosphorylation, the greater the contractile intensity of smooth muscle fibers. Inhibition of MLC phosphorylation can promote the relaxation of detrusor smooth muscle. Related studies have found that inhibiting the phosphorylation of MLC can reduce the sensitivity of myofilaments to Ca^2+^, which in turn causes the relaxation of rabbit detrusor muscles to alleviate the symptoms of interstitial cystitis [51]. The Western blot results revealed that EA can significantly reduce the expression of MLC phosphorylation in T2DM model rats. Our findings are consistent with the current evidence, which state that the downregulation of MLC phosphorylation can lead to detrusor smooth muscle relaxation. The contraction of detrusor smooth muscle depends on the increase in intracellular Ca^2+^, and the balance between MLCK and MLCP regulates the relationship between intracellular Ca^2+^ contractility [52]. Intracellular free Ca^2+^ binds with CAM to form Ca^2+^-CAM complex, which then binds with downstream MLCK and activates serine 19 of MLC 20. The p-MLC 20 myosin combines with actin to form a transmembrane bridge. ATPase in the head of myosin decomposes ATP to produce energy, which causes a cross-bridge swing and finally triggers myofilament contraction [53]. The contractile force of the bladder detrusor smooth muscle is correlated with the phosphorylation level of MLCK [52]. Our results show that EA can significantly increase the expression of MLCK phosphorylation in T2DM rats, suggesting that EA can regulate the excessive contractile activity of bladder detrusor smooth muscle.

The internal wall of the bladder detrusor smooth muscle is covered by mucosa, which also provides a sensory function to monitor the degree of bladder filling and composition of urine [54]. There is evidence that the gain of dynamic sensory structure system in the mucosa increases in diseases such as overactive bladder and bladder pain syndrome [54]. Furthermore, the mucosa layer can affect the spontaneous contraction of the bladder. Therefore, the state of bladder mucosa layer is very important for DBD. This study found that EA can reduce the infiltration of inflammatory cells in the mucosa layer of diabetic rats, thereby speculating that EA can improve the spontaneous contraction of the bladder detrusor by repairing the damaged bladder mucosa.

Micturition is a complex process, which mainly involves the activation and contraction of detrusor smooth muscle receptors [55, 56]. The storage and micturition function of bladder are related to detrusor. In bladder storage, an increase in urine volume distends the bladder detrusor. In micturition, the detrusor contracts rapidly to empty the urine. Smooth muscle contraction is essential for effective bladder emptying, and the cellular properties of detrusor muscle are very important for proper bladder function [57]. Three main structures are observed in a normal detrusor under TEM : SMCs, intercellular substance, and fibroblasts. As a functioning unit of the bladder, the shape of Mi in SMCs directly determines the contractile function of the bladder. In this study, it was observed that the Mi in detrusor SMCs of T2DM rats in the DM and sham EA group showed varying degrees of swelling, with the cristae of Mi broken, dissolving, or even disappearing and Mi autophagy. These ultrastructural changes suggest that the SMCs of T2DM rats lose their normal contractile function and the mutual coupling effect is decreased, leading to bladder detrusor dysfunction. After EA treatment, the swelling of Mi in the detrusor SMCs was reduced, suggesting that EA can repair the damage of the bladder detrusor and improve the stability of bladder function.

Previous studies have found that the phosphorylation level of MLC in diabetic animals is significantly higher [24]. Our results are consistent with these findings in that the MLC phosphorylation level and bladder weight increased in the DM group. After EA intervention, bladder weight and bladder wall thickness decreased, MLCK phosphorylation level increased, MLC phosphorylation level decreased, and the damage of the mucosa layer and detrusor SMCs improved, indicating that MLC and MLCK phosphorylation levels are two effective targets for EA treatment of DBD. As stated above, we suggest that the mechanism of EA on DBD is as follows: (1) EA increases MLCK phosphorylation and MLC dephosphorylation, leading to relaxation of bladder smooth muscle; (2) EA reduces the bladder hypertrophy to regulate the contractile function of detrusor smooth muscle and restore micturition reflex; (3) during the storage phase, EA may reduce the spontaneous contraction overactivity of detrusor by repairing the damaged bladder mucosa; and (4) TEM examination showed that EA could protect the ultrastructure of cell organelles, such as RER and Mi, in the bladder detrusor muscle of T2DM rats.

Cystometrogram is commonly used to assess bladder dysfunction. It is used to detect the effects of interventions on the three urodynamic parameters: Pv, max; emptying volume; and PVR volume [35, 58]. Furthermore, we chose in vitro ultrasound to observe the changes in bladder function in rats. But transabdominal ultrasound cannot accurately determine the bladder detrusor contractility/muscular tension. Therefore, this study has some limitations. Treatment of diabetes involves the regulation of specific biochemical factors affecting particular signal pathways, ultimately regulating cell apoptosis to repair a damaged structure [59]. Previous studies have confirmed that pituitary adenylate cyclase-activating polypeptide (PACAP) protein can regulate protein kinase A (PKA) through cyclic AMP (cAMP) and then promote the relaxation of SMCs [60, 61]. Therefore, further studies focusing on the effect of EA on PACAP-cAMP-PKA signal pathways in diabetic rats may explore new mechanisms of EA for DBD. Because other acupoints in the body may have similar effects on DBD, this study cannot clearly distinguish the mechanical effect of acupuncture from a specific acupoint. Therefore, it would be valuable to set an acupoint-control group in further research.

## 5. Conclusions

EA may alleviate bladder dysfunction in STZ–HFD-induced diabetic rats, reduce the bladder hypertrophy, and reverse the impairment in the mucosa layer and detrusor SMCs by regulating MLC and MLCK phosphorylation in the bladder detrusor.

## Figures and Tables

**Table 1:** General characteristics of the control, DM, EA, and sham EA groups.

Group	Body weight (g)	Serum glucose (mmol/L) (before intervention)	Serum glucose (mmol/L) (after intervention)	Bladder weight (mg)
Control	386.4 ± 12.5	3.7 ± 0.3	3.64 ± 0.25	120 ± 19.7
DM	225.7 ± 10.78^*∗*^	20.5 ± 2.5^*∗*^	20.6 ± 2.4^*∗*^	368 ± 29.5^*∗*^
EA	226.5 ± 9.5^*∗*^	21.3 ± 2.5^*∗*^	20.9 ± 2.6^*∗*^	216 ± 18.6^#^
Sham EA	225.1 ± 11.6^*∗*^	20.7 ± 2.6^*∗*^	20.5 ± 3.2^*∗*^	331 ± 25.6^*∗*^

Data were expressed as mean ± SEM. ^*∗*^*P* < 0.01 compared to the control group. ^#^*P* < 0.05 compared to the sham EA group.

**Table 2:** Urodynamic parameters of rats in each group.

Group	Volume threshold for micturition (mL)	PVR volume (mL)	Voided volume (mL)	*Pv*, max (mmHg)	*V*%
Control	0.57 ± 0.18	0.15 ± 0.07	0.64 ± 0.07	44.79 ± 6.13	19.37 ± 3.74
DM	2.55 ± 0.53^*∗*^	1.68 ± 0.36^*∗*^	0.96 ± 0.24	42.55 ± 7.66	61.85 ± 25.22^*∗∗*^
EA	1.66 ± 0.58^#^	0.82 ± 0.06^#^	1.29 ± 0.23^*∗*^	41.92 ± 6.01	38.49 ± 6.31^#^
Sham EA	2.16 ± 0.74	1.71 ± 0.25	1.31 ± 0.22^*∗*^	39.15 ± 5.04	56.18 ± 12.4

Data were expressed as mean ± SEM. ^*∗*^*P* < 0.05 compared to the control group; ^*∗∗*^*P* < 0.01 compared to the control group; ^#^*P* < 0.05 compared to the sham EA group.

## Data Availability

The data sets used and/or analyzed during the current study are available from the corresponding author on reasonable request.
